# Readmission Patterns to KMPT Acute Wards

**DOI:** 10.1192/bjo.2025.10515

**Published:** 2025-06-20

**Authors:** Olubunmi Olure, Valsraj Koravangattu

**Affiliations:** Kent and Medway NHS and Social Care Partnership Trust, Kent, United Kingdom

## Abstract

**Aims:** To identify patterns of readmission to acute wards and look for specific themes associated with readmissions – discharge planning, diagnosis, gender, social support, accommodation issues and any other associations.

To Identify improvement opportunities to align with the patient flow programme.

**Methods:** Data was gathered from KMPT Electronic patient record system. A total number of 12,602 admissions to all wards across KMPT between July 2019 and August 2024. The number of readmissions were extracted from this data.

12.4% of admissions were readmissions within 30 days following discharge. 95.38% of these 30-day readmissions were to Acute MH wards.

**Results:** Although KMPT has an improving picture in the number of 30-day readmissions compared with previous years, it is still 3% above the national average.

The 30-day readmissions have reduced over time from September 2019 to August 2024, an improving trend.

On an average KMPT currently has 24 readmissions per month. In order to achieve the national average, KMPT would have to reduce this from 24 per month to approximately 16 per month.

Patients aged between 25–35 had the highest 30-day readmission rate in the last year’s data.

There was a higher rate of readmissions for female patients.

The majority of 30-day readmissions have either not had their referral reason recorded but secondly indicate ‘In crisis’ as the reason for readmission.

Patients readmitted within 30 days of previous discharge were predominantly of cluster 8. They were also predominantly of ICD–10 code F603 at 18.75%.

**Conclusion:** This project is progressing under the Re-admissions pillar of the Patient Flow Improvement Project looking at both avoidable readmissions and high intensity user readmissions.

The Improvement project will look at data in greater detail and identify avoidable readmissions and high intensity users.

The purposeful admission pillar of the Improvement project will address the need to explicitly state what inpatient admission can achieve and the expected outcome from the admission.

